# The effectiveness of electronic payments on the market value of agricultural companies listed on the Iraq Stock Exchange

**DOI:** 10.12688/f1000research.177320.1

**Published:** 2026-03-05

**Authors:** Ahmad Hussein Battal, Abdulrazaq Shabeeb, Bha Aldan Abdulsattar Faraj, Wisam Al-Anezi, Faisal Ghazi Faisal

**Affiliations:** 1 Department of Economics, College of Administration and Economics, University of Anbar, Ramadi, Al Anbar Governorate, Iraq; 2 Department of Finance and Banking Sciences, College of Financial and Administrative Sciences, University of Al Maarif, Ramadi, 31001, Iraq; 3 Department of Finance and Banking, Al-Idrisi University College, Ramadi, 31001, Iraq

**Keywords:** Agricultural Companies, Iraq Stock Exchange, Electronic Payments, PMG_ARDL Model.

## Abstract

**Background:**

In light of the accelerating global shift toward financial digitalization, this research explores the impact of electronic payment instruments on the market value of agricultural companies listed on the Iraq Stock Exchange (ISX). The study addresses the necessity of understanding how digital transformation in financial transactions can mitigate challenges such as low liquidity and price volatility within the agricultural sector, ultimately aiming to enhance investment attractiveness and market performance.

**Methods:**

To achieve the study objectives, monthly data were collected for the period from June 2021 to May 2024, yielding a total of 144 observations. The sample comprised four listed agricultural firms: Al-Haditha for Animal Production, Middle East for Fish, Iraqi for Meat Production and Marketing, and Iraqi for Agricultural Products. The Pooled Mean Group - Autoregressive Distributed Lag (PMG-ARDL) model was employed to examine the relationship between the independent variables (mobile payments and electronic clearing) and the dependent variable (market value), while ensuring data stationarity and testing for long-term equilibrium.

**Results:**

Statistical findings revealed a significant positive impact of electronic payment methods on the market value of the sampled companies. Specifically, electronic checks (clearing) exhibited a more substantial influence; a 1% increase in their utilization leads to a 0.26% rise in market value. Meanwhile, a 1% increase in mobile payments contributes to a 0.032% appreciation in market value. These results underscore the efficacy of financial technology in optimizing market performance indicators.

**Conclusions:**

The study concludes that transitioning toward digital banking significantly enhances trading efficiency and fosters investor confidence, which is reflected positively in corporate valuation. The research recommends intensifying governmental and banking initiatives to strengthen digital infrastructure. Furthermore, it encourages large-scale agricultural enterprises to list on the ISX to deepen the Iraqi financial market and provide sustainable growth drivers for the national economy.

## 1- Introduction

In a time when continuous change has become the only constant, transitioning between changes necessitates searching for an appropriate way to measure fluctuations in financial and banking indicators in order to hedge against the financial risks that financial and non-financial companies may face. Since the changes occurring in the indicators of companies listed on the stock market are daily changes, their implications for the traded companies appear to be immediate ( Hussein et al. 2026). This drives us to build a standard model that simulates the real-time changes in financial indicators in a way that enables investors to make decisions closer to reality. The changes in the closing prices of agricultural companies listed on the Iraq Stock Exchange create a sense of anxiety for investors, which leads them to refrain from investing their funds in these companies, thereby affecting the necessary financing for those agricultural projects ( Battal et al. 2025). The paper aims to build a standard model that contributes to measuring the fluctuations in the closing prices of agricultural companies listed on the Iraq Stock Exchange. The importance of the paper lies in its assistance to investors and decision-makers in making appropriate investment decisions by referring to the results of the proposed model, which contributes to increasing profitability or reducing financial losses. The research problem emerges from the weak demand for the shares of agricultural companies listed on the Iraq Stock Exchange, as well as the continuous fluctuations in the prices of agricultural companies' shares, which reflects on the overall performance of the agricultural companies registered in the financial market. Naturally, this leads to the agricultural sector being weak and its competitiveness with imported equivalent goods being low ( Obed et al. 2025). The paper assumes that building a standard model for the fluctuations in the closing prices of shares of agricultural companies listed in the Iraqi financial market contributes to improving the performance of agricultural companies by monitoring the fluctuations in their prices and addressing them promptly. The researchers used both inductive and deductive methods to understand the reality of agricultural companies in the Iraq Stock Exchange and the closing price index of the listed companies. The paper included four agricultural companies listed on the Iraq Stock Exchange.

## 2- Previous studies

A study by ( Mustapha 2018) found that the development of electronic payment platforms, electronic banking services, electronic point of sale (POS) systems, and ATMs significantly contributed to the improvement of Nigerian banking performance. Additionally, a study by ( Ngaruiya, Bosire, and Kamau 2014) concluded that mobile financial transactions have a substantial impact on increasing sales revenues. The study by ( Augustin and Prosper 2023) indicated that electronic banking services contribute to the performance improvement of COGEBANQUE PLC, despite facing challenges such as weak networks and significant cybersecurity issues that threaten the confidentiality and integrity of banking information. Meanwhile, ( Awwad 2021) found that electronic payment methods have a significant and major impact on the financial performance indicators of banks (return on assets and equity), which helps reduce costs and, consequently, increase profits. In contrast, the study by ( Roy et al. 2021) concluded that ATMs do not contribute to the profitability of the banks in the sample, and point of sale systems are not significant for bank profitability; however, point of sale systems have a positive contribution to bank profitability. The study by ( Alsharaby 2024) found a long-term positive impact relationship of electronic payment methods on the financial performance indicators of the Iraqi banking sector by enhancing profitability and reducing costs. Torki concluded that all electronic payment indicators, including mobile banking, internet banking, card banking, POS systems, and ATMs, positively and significantly affect the financial performance of ten Islamic countries within the study sample ( Torki, Rezaei, and Razmi 2020).

We note that all previous studies did not address the impact of electronic transformation and electronic payments in Iraq and their effect on the financial performance indicators of companies listed on the Iraq Stock Exchange, in addition to dealing with several variables representing electronic payments (including mobile retail payments and electronic checks) and attempting to link them with the market value index of agricultural companies.

## 3- The relationship between electronic payments and the market value of agricultural companies

Electronic payments represent a broad field for employing financial technology to provide diverse financial services, such as payment, transfer, financing, insurance, investment, consulting, and others. Electronic payments aim to increase the efficiency, speed, and spread of financial service innovation, as well as to achieve customer satisfaction and alignment. Electronic payment methods contribute to enhancing banking performance and improving its financial indicators by improving the quality of financial transactions and executing them with high efficiency compared to traditional methods of completing financial transactions, in addition to reducing operational costs for managing cash transactions and checks. On the other hand, electronic payments provide opportunities for individuals who do not have traditional bank accounts to participate in the banking system, which contributes to bringing modern financial services to a wide customer base, leading to increased returns for financial and investment companies and improved performance indicators ( Ali and Faeq 2019). Therefore, the positive impact of electronic payments on financial performance indicators lies within the framework of the move towards financial inclusion as a result of technological advancement, increased job opportunities, and the introduction of new financial services such as investment and personal financing services, and the entry of new international customers, which contributes to enhancing the financial revenues of companies listed in the market ( Al-Anezi et al. 2025a). In addition to its role in enhancing financial performance indicators by reducing the costs of financial and banking services through minimizing the need for traditional paper management, which leads to improved efficiency in banking operations, this contributes to eliminating excess costs and strengthening the financial resources of management, thereby enhancing the financial performance efficiency of the institution. Empirical studies have indicated a positive effect between the development of electronic payments and the financial performance of investment companies in general and agricultural companies in particular, by improving the collection process and reducing its durations through transferring those funds online and increasing the financial liquidity of agricultural companies, which increases their investment activity, leading to increased profits, which results in higher closing prices and, consequently, an increase in their market value.

From here, the importance of electronic payment methods emerges in enhancing financial performance in general and the market value of agricultural companies in particular, through their impact on the dimensions of financial performance. The effectiveness that most agricultural companies strive to achieve efficiently means rationalizing financial expenditures and operating in an attractive investment environment. All of this points to the concept of effectiveness, which is working to achieve set goals with the highest levels of discipline and rationalization. Therefore, electronic payments contribute to achieving efficiency and effectiveness by eliminating traditional administrative units and the financial expenses that burden the company's budget, as long as there is a transition from traditional management and trading methods to modern management based on financial technology and technical developments in the field of currency trading and transfer. Moreover, employing financial technology and using electronic payments in commercial transactions will encourage unregistered agricultural companies in the market to register and offer their shares for daily trading, which will positively reflect on the number of shares traded, and thus the closing prices of profitable companies' shares will rise, leading to an increase in the market value index. On the other hand, daily electronic transactions contribute to creating added economic value for the overall economy by encouraging foreign agricultural companies to engage in commercial and investment dealings with local agricultural companies, thereby facilitating the transfer of modern agricultural technology and the development of its various means, improving its tools and trading indicators. This will revitalize the real and financial agricultural sector, and consequently, the contribution of that sector to the formation of the gross domestic product will increase in general ( Hammad et al. 2024).

The mechanism of the impact of the spread of electronic payment tools and methods on the stocks of agricultural companies can be explained by the fact that the spread of electronic payment methods (electronic cards, electronic checks, financial transfers via mobile) directly contributes to accelerating the processes of buying and selling stocks of all kinds, especially the stocks of agricultural companies ( Zaidan, Rustum, and Mahdi 2024). This leads to an increase in the prices of the stocks of agricultural companies listed in the financial market, and the rise in the market value of agricultural companies will reflect an increase in their competitiveness in the market. Consequently, their profits and sales will increase, which will contribute to strengthening and supporting the agricultural sector in general.

## 4- Materials and methods

The paper used the (PMG-ARDL) model to measure the effect of electronic payments on the market value index of a group of agricultural companies listed on the Iraq Stock Exchange, as detailed in the following steps:

### 4-1: Unit root for heterogeneous panel data

We can use several methods to test whether there is a unit root for panel data in our study, and we used the (Breitung & Im, Pesaran, & Shin) tests, employing a unit root test for panel data. Moreover, this test provides t-test statistics that follow the normal distribution of residuals, with each cross-section receiving a separate estimate, allowing for the specification of parameter values, residual variance, and lag length. Furthermore, the test can be conducted using high power, with little distortion, as well as for small samples. The Augmented Dickey-Fuller (ADF) test is as follows ( Pedroni 2004):

∆Yit=ai+piYt−1+∑£jYt−1+βit+ϵt (1)

### 4-2: Co-integration test for heterogeneous panel data

We can determine whether there is a long-term joint integration relationship between electronic payments and the market value of agricultural companies listed on the Iraq Stock Exchange by applying the Pedroni cointegration test, after ensuring that there is no unit root in the panel data ( Muhammed and Alhiyali 2018). The cointegration test allows for multiple regression and estimates the residuals from the cointegration regressions, providing seven statistical tests. It permits joint integration to vary across the existing sections in the panel, in addition to the heterogeneity between cross-sectional units (panel data). The estimated residuals from the long-term regression are as follows ( Fahad, Battal, and Yaseen 2023):

$$Y\mathrm{it}=\mathrm{ai}+\mathrm{\pi i}+\sum_{m=1}^{m}\mathrm{\beta mt}.\text{Xmit}+\mathrm{\mu ti}$$ (2)

The intersection and regression transactions differ according to the cross-sectional units. Pedroni used critical values to test the seven statistics using a Monte Carlo simulation, suggesting that if the values of the statistics exceed the critical values, the null hypothesis should be rejected and the alternative hypothesis accepted, indicating the absence of cointegration ( Al-Anezi et al. 2025b).

### 4-3: Estimation of the (PMG-ARDL) model

Pesaran and Smith (1995) introduced the mean group (MG) as a simple estimator for panel data with a set of observations for time series across several groups (N). When considering the estimation of the (ARDL p, q) model, which relies on a panel data set with time series (i = 1,2 …. N) and time periods (t = 1, … T), T contains a sufficient number of observations to estimate the model and determine the degree of effect ( Asteriou and Hall 2006).

$$Y\mathrm{it}=\sum_{j=1}^{p}⋋\mathrm{ij}*Yi,t-j+\sum_{j=0}^{q}Ҩ\mathrm{ij}*Xi+y1\mathrm{dt}+\mathrm{\epsilon it}$$ (3)

Since xit and dt are (k × 1) and (s × 1) rebound factors, while ⋋ij∗s represents the residuals and Ҩij∗s and (ϵi∗ s are (k × 1)) and (s × 1) vectors of unspecified parameters to be estimated. As in the following equation ( KAMACI, CEYHAN, and PEÇE 2019).

$$∆Y\mathrm{it}=\sum_{k=1}^{p-1}⋋\mathrm{ik}∆Yi,t-1+\sum_{k=0}^{q-1}\begin{array}{c} {}*\\ ⋋ \end{array}\mathrm{ik}∆Yi,t-1+Ҩi\left(Yi,t-1+\text{βiXit}\right)+\mathrm{\omega i}+\mathrm{\epsilon it}$$ (4)

### 4-4: Hiaso test for homogeneity of variance

Hiaso tests are one of the essential stages in determining the appropriate methodology for estimating panel data. In the case of complete homogeneity of intercepts and slopes, the panel data method is rejected in favor of the Ordinary Least Squares (OLS) method for estimating a pooled model. However, if the slopes are only homogeneous, the most suitable estimation model would be fixed or random effects, respectively. In the case of complete heterogeneity of intercepts and slopes, one must rely on one of the dynamic models for estimation.

### 4-5: Descriptive analysis of variables

Most electronic payment methods in the financial sector in Iraq witnessed significant development after 2020. This is due to the government’s approach to reform the Iraqi banking sector and improve its poor reality, leading to structural improvements and amendments to the banking law and the financial sector in general. Amendments were made to the operational mechanism in the Iraqi stock market to enable it to keep pace with developments in emerging financial markets ( Dawood et al. 2025). The advancements in electronic payment methods have significantly contributed to improving the market value index of agricultural companies listed in the market. This is reflected in customers' desire to use ATM cards or online payments or the massive expansion in mobile phone usage, which has impacted financial and real investment methods and improved the financial results of agricultural companies. Consequently, their profits increased, leading to a rise in the closing prices of their shares, which resulted in an increase in the number of traded shares, indicating a rise in the market value index of the agricultural companies listed in the market. The annual statistical bulletins issued by the Central Bank for electronic payment variables for the monthly period 2021-2024 were relied upon, while the stock price data of agricultural companies were obtained from the annual reports of the Iraq Stock Exchange for the monthly period 2021-2024.

**Figure 1.**
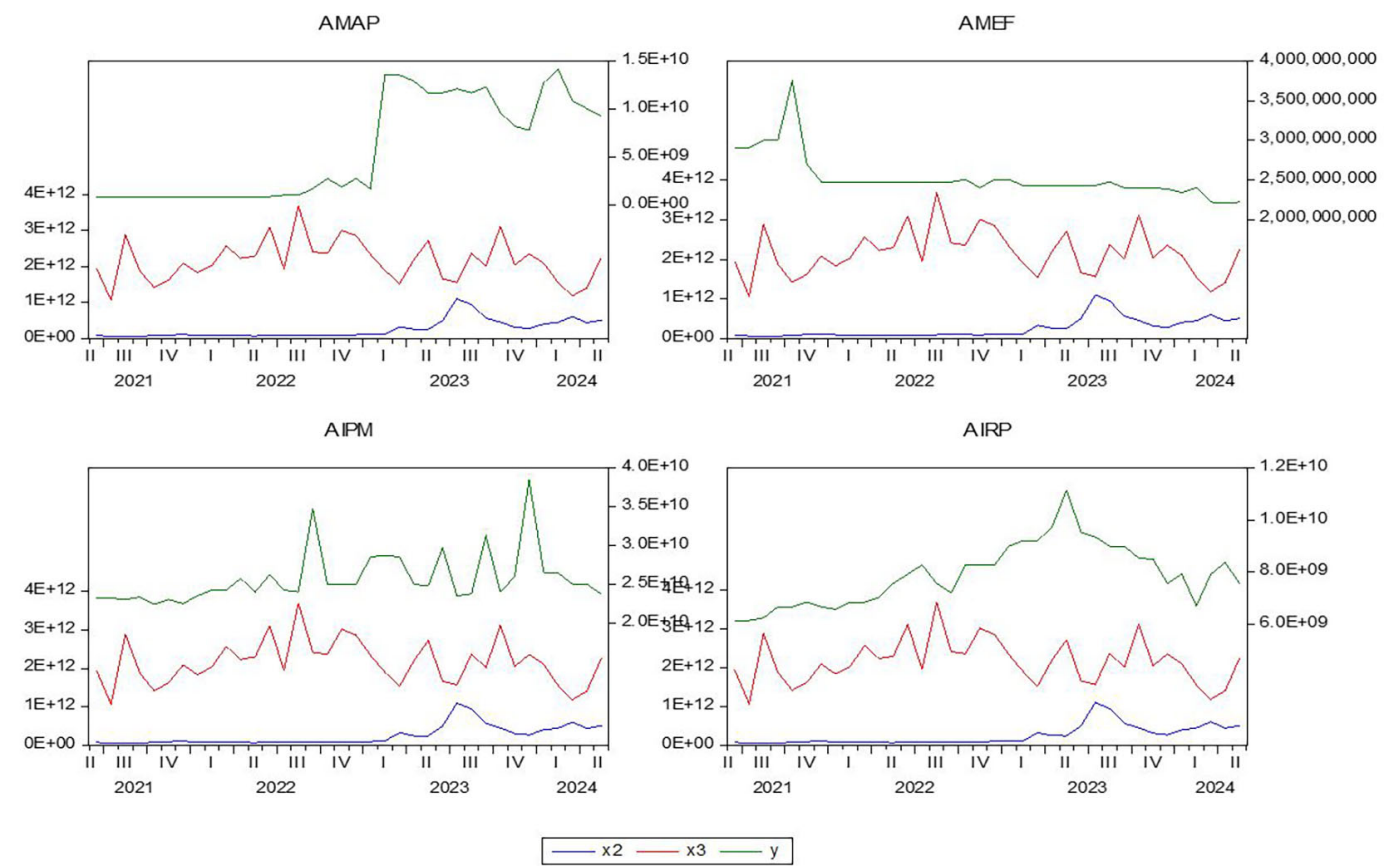
Time paths of research variables.

Figure 1 shows Most electronic payment methods in the financial sector witnessed a remarkable development after the year 2020 in Iraq. This is due to the government’s approach to reforming the Iraqi banking sector and improving its declining reality. It worked to introduce structural improvements and amend the banking law and the financial sector in general, and made amendments to the mechanism of work in the Iraqi Stock Exchange. In a way that enables it to keep pace with developments taking place in emerging financial markets.

## 5- Descriptive statistics variables

Table 1 shows the descriptive statistics for the study variables consisting of 144 observations. It is evident that the mean of the market value variable (Y1) reached (10,500) billion dinars, while the independent variables recorded their highest value equal to the market value at (39) billion dinars, whereas the lowest levels for those companies were (1) billion dinars. As for the mobile retail payment variable (X1), its highest value reached (1,100,000) monetary units, while its lowest value was (59,300) monetary units. The total electronic checks variable (X2) recorded its highest value during the study period at (3,680,000) monetary units, while the lowest recorded value was (1,070,000) monetary units.

**Table 1.** Results of descriptive statistics for study variables.

	Market value Y	Mobile payment (X2)	Electronic checks (X3)
**Mean**	10500	257000	2150000
**Median**	8	107000	2100000
**Maximum**	39	1100000	3680000
**Minimum**	1	59300	1070000
**Std. Dev.**	10	252000	576000
**Skewness**	0.911238	1.661842	0.434285
**Kurtosis**	2.530615	5.459056	3.003879
**Jarque-Bera **	21.25046	102.563	4.526566
**Probability**	0.000024	0.000	0.104008
Observations	144	144	144

Source: Outputs of the Econometric program EVIEWS 13.

## 6- Results and discussion

After reviewing the theoretical and analytical aspects of the behavior of electronic payment variables in the market value index of a sample of agricultural companies listed on the Iraq Stock Exchange, it is necessary to apply a standard model to determine the extent to which electronic payments impact the dependent variable.

Table 2 shows the agricultural companies listed in the Iraqi market on which the study was conducted.

**Table 2.** Variable abbreviations.

Abbreviations	Company	Abbreviations	Company
**AIPM**	Iraqi Company for Meat Production and Marketing	**AMAP**	For the Modern Company for Animal Production
**AIRP**	Iraqi Company for Agricultural Products	**AMEF**	Middle East Fish Company

Source: Outputs of the Econometric program EVIEWS 13.

### 7-1: Description of the econometric model

### 7-2: Analysis of PMG-ARDL model results

The PMG-ARDL model was applied to the data of agricultural companies listed on the Iraq Stock Exchange, and the estimation results of the relationship are as follows:

**7-2-1: Results of the panel data stationarity test**

Table 3 shows the results of the panel data stationarity test as indicated below.

**Table 3.** Stationarity tests for research variables.

	TEST	Level		First difference	
		Statistic	Prob.	Statistic	Prob.
**Y**	Levin, Lin & Chu t*	-1.530	0.063	-13.163	0.000
	Im, Pesaran and Shin W-stat	-1.582	0.057	-13.293	0.000
	ADF - Fisher Chi-square	15.450	0.051	116.158	0.000
	PP - Fisher Chi-square	25.867	0.001	126.626	0.000
**X2**	Levin, Lin & Chu t*	-1.639	0.051	-13.163	0.000
	Im, Pesaran and Shin W-stat	-0.313	0.377	-13.293	0.000
	ADF - Fisher Chi-square	6.551	0.586	116.158	0.000
	PP - Fisher Chi-square	5.920	0.656	126.626	0.000
**X3**	Levin, Lin & Chu t*	-8.259	0.000	-19.923	0.000
	Im, Pesaran and Shin W-stat	-7.663	0.000	-19.546	0.000
	ADF - Fisher Chi-square	64.335	0.000	153.746	0.000
	PP - Fisher Chi-square	65.828	0.000	110.202	0.000

Source: Outputs of the Econometric program EVIEWS 13.

It is evident from Table 3 that the time series of variable X1 is unstable at its original level according to various stationarity tests for panel data at the 5% level, as is variable Y, which necessitated taking the first differences, making it stationary at the first difference. Meanwhile, variable X2 is stationary at the original level. Therefore, it is necessary to employ the Panel ARDL Model for the panel data due to the differences in the degrees of stationarity of the variables.

**7-2-2: Hiaso test for homogeneity of cross sections**

Table 4 shows the results of the Hiaso test for the homogeneity of cross sections as follows:

**Table 4.** Results of the Hiaso test for homogeneity.

Hypotheses	F-stat	P-value
*H _1_*	199.9492	0.001
*H _2_*	9.370854	0.001
*H _3_*	426.0463	0.001

Source: Outputs of the Econometric program EVIEWS 13.

It is clear from Table 4 that the panel data for the agricultural companies in the study sample lack homogeneity and therefore static models cannot be applied because they give us inefficient estimates, and this does not help us in arriving at solutions to the research problem aimed at measuring the impact of electronic payments on the market value index of agricultural companies listed in Iraq Stock Exchange.

First Hypothesis: Assuming the existence of complete homogeneity of segments and coefficients, and after conducting the estimation process, the following was revealed:

F1=199.9492,prob.F1=0.001

It is clear from the above results that the calculated value of F1 is greater than its tabulated value at a significance level of less than 5%. Therefore, we reject the null hypothesis and accept the alternative hypothesis which states that there is no homogeneity of the overall data for the panel data model. This leads us to test the second hypothesis as follows:

Second Hypothesis: Assuming the existence of homogeneity of coefficients only, and after the estimation process, the following was revealed:

F2=9.370854,Prob.F2=0.001

As evident from the above results, the calculated value of F2 is greater than its tabulated value at a significance level of less than 5%. Therefore, we reject the null hypothesis indicating homogeneity of the slope coefficients and accept the alternative hypothesis which states that there is no homogeneity of the overall data for the panel data model. This leads us to test the third hypothesis as follows:

Third Hypothesis: Assuming homogeneity of constants only, and after the estimation process, the following was revealed:

F3=426.0463,Prob.F3=0.001

It is clear from the above results that the calculated value of F3 is greater than its tabulated value at a significance level of less than 5%. Therefore, we reject the null hypothesis indicating homogeneity of the slope coefficients and accept the alternative hypothesis which states that there is no homogeneity of the overall data for the panel data model.

Table 4 shows that the panel data for the agricultural companies in the sample study lacks homogeneity; therefore, static models cannot be applied as they provide us with inefficient estimators, which does not help us in reaching solutions to the research problem aimed at measuring the impact of electronic payments on the market value index of agricultural companies listed on the Iraq Stock Exchange.

**7-2-3: Cointegration test for panel data**

Table 5 shows the results of the (Pedroni) test, and based on the extracted results, it was found that there are (6) statistically significant statistics out of (8) statistics. Therefore, the null hypothesis was rejected and the alternative hypothesis was accepted for four tests: (Panel PP-Statistic), (Panel ADF-Statistic), (Group PP Statistic), and (Group ADF-Statistic) at confidence levels of 95% and 99%. Consequently, the results of the different cointegration tests indicate that the variables of electronic payments (retail payment via mobile and electronic checks) are jointly cointegrated with the market value of agricultural companies listed on the Iraq Stock Exchange.

**Table 5.** Results of the cointegration test for panel data.

Pedroni residual cointegration test
Series: Y X2 X3
Sample: 2021M06 2024M05
Included observations: 144
Cross-sections included: 4
Null Hypothesis: No cointegration
Trend assumption: No deterministic trend

Alternative hypothesis: common AR coefs. (within-dimension)				
Weighted				
	Statistic	Prob.	Statistic	Prob.
Panel v-Statistic	0.060745	0.4758	-0.491332	0.6884
Panel rho-Statistic	-3.889090	0.0001	-2.249976	0.0122
Panel PP-Statistic	-4.426724	0.0000	-2.823369	0.0024
Panel ADF-Statistic	-4.352852	0.0000	-2.862183	0.0021

Source: Outputs of the Econometric program EVIEWS 13.

**7-2-4: Analysis of Kao residual cointegration test results**

Table 6 shows the results of the (Kao) test regarding the extent to which there is a cointegration relationship between electronic payments and market value. This is evident from the Kao value of (-2.217846), which was significant at the significance level (5%).

**Table 6.** Results of Kao residual cointegration test.

Series: Y X2 X3
Sample: 2021M06 2024M05
Null Hypothesis: No cointegration
Trend assumption: No deterministic trend

	t-statistic	Prob.
ADF	-2.217846	0.0133

Source: Outputs of the Econometric program EVIEWS 13.

**7-2-5: Estimation of panel ARDL model results**

Table 7 shows that the error correction coefficient (COINTEQ01) reached (-0.878165) with a negative and significant value at a 10% significance level, indicating the presence of a long-term cointegration relationship. From this, we conclude the following:

• There is a positive effect of the mobile retail payment variable (X2) on the market value index, as a 1% increase in mobile retail payments leads to a 0.032% increase in market value, and this relationship is significant at a 5% significance level.
• There is a positive effect of the electronic sukuk variable (X3) on the market value index, as a 1% increase in the value of electronic sukuk leads to a 0.2627% increase in market value, and this relationship is significant at a 5% significance level.

**Table 7.** Results of panel ARDL model.

Dependent variable: DLOG(Y)
Method: ARDL
Sample: 2022M01 2024M05
Included observations: 116
Fixed regressors: C @TREND
Number of models evalulated: 32
Selected Model: ARDL(7, 4, 4)

Variable	Coefficient	Std. error	t-statistic	Prob.*
***Long Run Equation***				
LOG(X2)	0.032945	0.009378	3.513036	0.0008
LOG(X3)	0.262701	0.046753	5.618889	0.0000
***Short Run Equation***				
COINTEQ01	-0.878165	0.507258	-1.731199	0.0876
DLOG(Y(-1))	-0.005098	0.195689	-0.026053	0.9793
DLOG(Y(-2))	0.152375	0.151759	1.004056	0.3186
DLOG(Y(-3))	-0.022868	0.122702	-0.186372	0.8527
DLOG(Y(-4))	-0.156284	0.173756	-0.899445	0.3713
DLOG(Y(-5))	0.019263	0.314091	0.061329	0.9513
DLOG(Y(-6))	0.035233	0.347325	0.101440	0.9195
DLOG(X2)	-0.013507	0.043125	-0.313200	0.7550
DLOG(X2(-1))	-0.392770	0.275708	-1.424588	0.1585
DLOG(X2(-2))	0.058006	0.038461	1.508168	0.1358
DLOG(X2(-3))	0.010842	0.029911	0.362485	0.7180
DLOG(X3)	-0.566115	0.229992	-2.461455	0.0162
DLOG(X3(-1))	-0.521758	0.225691	-2.311819	0.0236
DLOG(X3(-2))	-0.473927	0.257207	-1.842594	0.0694
DLOG(X3(-3))	-0.282573	0.182897	-1.544984	0.1266
C	13.02076	8.119829	1.603576	0.1131
@TREND	0.006267	0.007262	0.863016	0.3909
Mean dependent var	0.021066	S.D. dependent var		0.243603
S.E. of regression	0.171149	Akaike info criterion		-1.583560
Sum squared resid	2.167601	Schwarz criterion		-0.139900
Log likelihood	184.0163	Hannan-Quinn criter.		-0.996938

Source: Outputs of the Econometric program EVIEWS 13.

**7-2-6: Choosing the best ARDL model**

Table 8 shows the results of the (ACI) test for selecting the best estimated model. After calculating the results, it was found that the best model is (ARDL(7, 4, 4)). This model indicates that seven lag periods were estimated for the dependent variable, four lag periods for the first independent variable (retail payment via mobile), and four lag periods for the second independent variable (electronic check).

**Table 8.** Results of estimating the best ARDL model.

Model selection criteria table
Dependent Variable: LOG(Y)
Date: 07/24/24 Time: 13:55
Sample: 2021M06 2024M05
Included observations: 144

Model	LogL	AIC*	BIC	HQ	Specification
28	177.949770	-1.927674	-0.228613	-1.238311	ARDL(7, 4, 4)
31	172.488783	-1.901585	-0.299613	-1.251614	ARDL(8, 3, 3)
27	168.388837	-1.899801	-0.394917	-1.289222	ARDL(7, 3, 3)
32	179.760458	-1.888580	-0.092429	-1.159824	ARDL(8, 4, 4)
11	149.573738	-1.849531	-0.733005	-1.396521	ARDL(3, 3, 3)
7	145.450370	-1.847328	-0.827891	-1.433710	ARDL(2, 3, 3)
12	157.349851	-1.845533	-0.534828	-1.313738	ARDL(3, 4, 4)
19	156.852523	-1.836652	-0.525947	-1.304858	ARDL(5, 3, 3)
16	160.731414	-1.834490	-0.426695	-1.263303	ARDL(4, 4, 4)
8	152.437059	-1.829233	-0.615618	-1.336831	ARDL(2, 4, 4)
3	140.274956	-1.826339	-0.903991	-1.452113	ARDL(1, 3, 3)
20	164.229419	-1.825525	-0.320642	-1.214946	ARDL(5, 4, 4)
15	151.830426	-1.818400	-0.604785	-1.325998	ARDL(4, 3, 3)
23	159.643796	-1.815068	-0.407274	-1.243881	ARDL(6, 3, 3)
2	131.110870	-1.805551	-1.077382	-1.510110	ARDL(1, 2, 2)
24	166.055364	-1.786703	-0.184730	-1.136732	ARDL(6, 4, 4)
6	133.765838	-1.781533	-0.956274	-1.446699	ARDL(2, 2, 2)
4	145.577032	-1.778161	-0.661635	-1.325151	ARDL(1, 4, 4)
1	121.055473	-1.768848	-1.234857	-1.552191	ARDL(1, 1, 1)
5	124.627755	-1.761210	-1.130130	-1.505161	ARDL(2, 1, 1)
9	125.474358	-1.704899	-0.976730	-1.409458	ARDL(3, 1, 1)
10	131.884615	-1.676511	-0.754163	-1.302285	ARDL(3, 2, 2)
13	127.442800	-1.668621	-0.843363	-1.333788	ARDL(4, 1, 1)
14	133.371824	-1.631640	-0.612203	-1.218022	ARDL(4, 2, 2)
26	144.862390	-1.622543	-0.311838	-1.090748	ARDL(7, 2, 2)
17	128.216891	-1.611016	-0.688668	-1.236790	ARDL(5, 1, 1)
30	147.847669	-1.604423	-0.196629	-1.033236	ARDL(8, 2, 2)
18	135.350906	-1.595552	-0.479026	-1.142542	ARDL(5, 2, 2)
21	130.828050	-1.586215	-0.566778	-1.172597	ARDL(6, 1, 1)
22	138.425250	-1.579022	-0.365407	-1.086620	ARDL(6, 2, 2)
25	131.897235	-1.533879	-0.417353	-1.080869	ARDL(7, 1, 1)
29	133.020789	-1.482514	-0.268899	-0.990112	ARDL(8, 1, 1)

Source: Outputs of the Econometric program EVIEWS 13.

**7-2-7: Results of the short-term cointegration relationship**

**7-2-7-1: The short-term relationship of the Modern Animal Production Company (AMAP)**

Table 9 shows the results of estimating the short-term relationship between electronic payments indicators and the market value of the Modern Animal Production Company (AMAP) over the study period. The table shows the existence of a short-term cointegration relationship, as evidenced by the value of the error correction coefficient (COINTEQ01), which reached (-0.396620), which was significant at a significance level of (5%).

**Table 9.** Results of estimating the short-term relationship between electronic payments and the market value of the modern animal production company.

Variable	Coefficient	Std. error	t-statistic	Prob. *
COINTEQ01	-0.396620	0.037205	-10.66050	0.0018
DLOG(Y(-1))	-0.143019	0.051591	-2.772196	0.0694
DLOG(Y(-2))	0.574621	0.052376	10.97109	0.0016
DLOG(Y(-3))	-0.115508	0.032966	-3.503862	0.0394
DLOG(Y(-4))	0.272515	0.049896	5.461603	0.0121
DLOG(Y(-5))	0.882374	0.055657	15.85381	0.0005
DLOG(Y(-6))	0.543634	0.043808	12.40940	0.0011
DLOG(X2)	-0.096101	0.079514	-1.208605	0.3134
DLOG(X2(-1))	-1.202167	0.096007	-12.52160	0.0011
DLOG(X2(-2))	0.089944	0.065329	1.376791	0.2623
DLOG(X2(-3))	0.084865	0.061014	1.390922	0.2585
DLOG(X3)	-1.074318	0.066178	-16.23367	0.0005
DLOG(X3(-1))	-1.082323	0.197651	-5.475938	0.0120
DLOG(X3(-2))	-1.201581	0.247935	-4.846351	0.0168
DLOG(X3(-3))	-0.815988	0.106584	-7.655846	0.0046
C	4.835909	4.535751	1.066176	0.3645
@TREND	0.027537	0.000797	34.55661	0.0001

Source: Outputs of the Econometric program EVIEWS 13.

Table 9 shows the results of estimating the short-term relationship between electronic payment indicators and the market value index of the Modern Animal Production Company (AMAP) during the study period. It is clear from the table that there is a short-run cointegration relationship, and this is evident through the value of the error correction factor (COINTEQ01), which reached (-0.396620), which was significant at the significance level (5%).

**7-2-7-2: The short-term relationship of the Middle East Fisheries Company (AMEF)**

Table 10 shows the results of estimating the short-term relationship between electronic payments indicators and the market capitalization index of the Middle East Fisheries Company (AMEF) over the study period. The table shows a short-term cointegration relationship, as evidenced by the value of the error correction coefficient (COINTEQ01), which reached (-0.628304), which was significant at a significance level of (5%).

**Table 10.** Results of estimating the short-term relationship between electronic payments and the market value of the Middle East Fish Company.

Variable	Coefficient	Std. error	t-statistic	Prob. *
COINTEQ01	-0.628304	0.028022	-22.42145	0.0002
DLOG(Y(-1))	-0.316750	0.008956	-35.36874	0.0000
DLOG(Y(-2))	-0.142636	0.004425	-32.23342	0.0001
DLOG(Y(-3))	-0.179541	0.002211	-81.18697	0.0000
DLOG(Y(-4))	-0.089323	0.001672	-53.43382	0.0000
DLOG(Y(-5))	-0.039238	0.001068	-36.75390	0.0000
DLOG(Y(-6))	-0.061481	0.000572	-107.4974	0.0000
DLOG(X2)	0.006992	5.25E-05	133.0754	0.0000
DLOG(X2(-1))	0.033629	6.73E-05	499.6075	0.0000
DLOG(X2(-2))	0.002384	7.11E-05	33.54361	0.0001
DLOG(X2(-3))	-0.008354	4.50E-05	-185.8271	0.0000
DLOG(X3)	-0.106163	0.001210	-87.71432	0.0000
DLOG(X3(-1))	-0.035113	0.000914	-38.43546	0.0000
DLOG(X3(-2))	-0.004935	0.000409	-12.06347	0.0012
DLOG(X3(-3))	-0.031094	0.000118	-263.6099	0.0000
C	8.374661	8.220384	1.018768	0.3833
@TREND	-0.001758	8.44E-07	-2081.606	0.0000

Source: Outputs of the Econometric program EVIEWS 13.

Table 10 shows the results of estimating the short-term relationship between electronic payment indicators and the market value index of the Middle East Fisheries Company (AMEF) during the study period. It is clear from the table that there is a short-run cointegration relationship, and this is evident through the value of the error correction factor (COINTEQ01), which reached (-0.628304), which was significant at the significance level (5%).

**7-2-7-3: The short-term relationship of the Iraqi company for meat production and marketing (AIPM)**

Table 11 shows the results of estimating the short-term relationship between electronic payments indicators and the market value of the Iraqi Company for Meat Production and Marketing (AIPM) over the study period. The table shows a short-term cointegration relationship, as evidenced by the value of the error correction coefficient (COINTEQ01), which reached (-2.367686), which was significant at a significance level of (5%).

**Table 11.** results of estimating the short-term relationship between electronic payments and the market value of the Iraqi company for meat production and marketing.

Variable	Coefficient	Std. error	t-statistic	Prob. *
COINTEQ01	-2.367686	0.653224	-3.624618	0.0361
DLOG(Y(-1))	0.567770	0.553326	1.026105	0.3803
DLOG(Y(-2))	0.050337	0.409491	0.122925	0.9099
DLOG(Y(-3))	-0.139522	0.334398	-0.417234	0.7046
DLOG(Y(-4))	-0.563932	0.269186	-2.094950	0.1272
DLOG(Y(-5))	-0.619425	0.186171	-3.327188	0.0448
DLOG(Y(-6))	-0.910560	0.141218	-6.447899	0.0076
DLOG(X2)	0.098620	0.005798	17.00886	0.0004
DLOG(X2(-1))	-0.232044	0.006043	-38.40076	0.0000
DLOG(X2(-2))	-0.011874	0.004000	-2.968297	0.0591
DLOG(X2(-3))	-0.057713	0.004640	-12.43691	0.0011
DLOG(X3)	-0.828141	0.034704	-23.86266	0.0002
DLOG(X3(-1))	-0.654283	0.041641	-15.71237	0.0006
DLOG(X3(-2))	-0.418676	0.026354	-15.88680	0.0005
DLOG(X3(-3))	-0.224968	0.008980	-25.05219	0.0001
C	37.04709	216.6920	0.170967	0.8751
@TREND	0.003405	1.74E-05	196.2380	0.0000

Source: Outputs of the Econometric program EVIEWS 13.

Table 11 shows the results of estimating the short-term relationship between electronic payments indicators and the market value index of the Iraqi Company for Meat Production and Marketing (AIPM) during the study period. It is clear from the table that there is a short-run cointegration relationship, and this is evident through the value of the error correction factor (COINTEQ01), which reached (-2.367686), which was significant at the significance level (5%).

**7-2-7-4: Short-term relationship of the Iraqi agricultural products company AIPR**

Table 12 shows the results of estimating the short-term relationship between electronic payments indicators and the market value of the Iraqi Company for Meat Production and Marketing (AIPM) over the study period. The table shows a short-term cointegration relationship, as evidenced by the value of the error correction coefficient (COINTEQ01), which reached (-0.120049), which was significant at a significance level of (10%).

**Table 12.** Results of estimating the short-term relationship between electronic payments and the market value of the Iraqi company for agricultural products.

Variable	Coefficient	Std. error	t-statistic	Prob. *
COINTEQ01	-0.120049	0.058564	-2.049878	0.1328
DLOG(Y(-1))	-0.128394	0.074301	-1.728018	0.1824
DLOG(Y(-2))	0.127176	0.085149	1.493575	0.2321
DLOG(Y(-3))	0.343099	0.104883	3.271254	0.0467
DLOG(Y(-4))	-0.244394	0.092624	-2.638557	0.0778
DLOG(Y(-5))	-0.146659	0.099460	-1.474564	0.2368
DLOG(Y(-6))	0.569338	0.061510	9.256095	0.0027
DLOG(X2)	-0.063538	0.002516	-25.25265	0.0001
DLOG(X2(-1))	-0.170498	0.003712	-45.93070	0.0000
DLOG(X2(-2))	0.151568	0.002524	60.04020	0.0000
DLOG(X2(-3))	0.024572	0.001952	12.58902	0.0011
DLOG(X3)	-0.255838	0.004872	-52.51463	0.0000
DLOG(X3(-1))	-0.315311	0.007228	-43.62096	0.0000
DLOG(X3(-2))	-0.270517	0.007927	-34.12635	0.0001
DLOG(X3(-3))	-0.058242	0.004542	-12.82339	0.0010
C	1.825375	11.72476	0.155685	0.8862
@TREND	-0.004116	1.94E-05	-212.3988	0.0000

Source: Outputs of the Econometric program EVIEWS 13.

Table 12 shows the results of estimating the short-term relationship between electronic payments indicators and the market value index of the Iraqi Company for Meat Production and Marketing (AIPM) during the study period. It is clear from the table that there is a short-run cointegration relationship, and this is evident through the value of the error correction factor (COINTEQ01), which reached (-0.120049), which was significant at the significance level (10%).

### 7-3- Model diagnostic tests

Model diagnostic tests are used to assess the validity and verify the underlying assumptions. These tests also help detect issues such as multicollinearity or heteroscedasticity to ensure the accuracy and reliability of the results. The table below shows the results of the heteroscedasticity test.

Table 13 shows that the joint test accepts the null hypothesis that the model does not suffer from the problem of heteroscedasticity instability, whether at the overall level or at the level of the four companies, because the significance is greater than 5%.

**Table 13.** Heteroskedasticity robust standard error estimates.

Statistics	Max|z|	Prob.
Fisher Combined	15.108	0.057
Cross-section Joint Tests		

Cross-section	Max|z|	Prob.*
AMAP	1.766	0.275
AMEF	2.350	0.073
AIPM	2.214	0.103
AIRP	1.810	0.253

As shown in Table 14 and according to the correlation test between cross-sections, the model residuals do not include the problem of correlation between cross-sections, and according to the four tests, the significance level is greater than 5%.

**Table 14.** Cross-section dependence (correlation) test.

Null hypothesis: No cross-sectional dependence (correlation)
Cross-sections included: 4
Total panel observations: 116

Test	Statistic	Prob.
Breusch-Pagan LM	6.191	0.402
Pesaran scaled LM	0.055	0.956
Bias-corrected scaled LM	-0.016	0.987
Pesaran CD	1.693	0.091

## 8- Conclusions

The research hypothesis, which indicates a long-term, complementary relationship between electronic payments and the market capitalization index (MCI) of a sample of agricultural companies listed on the Iraq Stock Exchange, is confirmed. The development of electronic payments has significantly improved the market capitalization index of the companies in the study sample. This is evident in the role of these modern financial technologies in reducing companies' expenses and time and speeding up financial transactions. This has encouraged many companies to list their shares on the market and offer them for trading, which has positively impacted the market capitalization index. Electronic payments contribute to further activating the role of agricultural companies by enabling them to obtain the necessary financing quickly. This is achieved by taking advantage of the advantages of electronic transactions and the reduced cost and time of financing. This will open the way for new companies to enter the financial market. An increase in the electronic checks index by one unit leads to an increase in the market value index by (0.26%), while if the value of retail payment via mobile increases by one unit, it leads to an increase in the market value by (0.032%). This indicates the role of electronic checks in influencing the performance of agricultural companies.

The study recommends that agricultural companies listed on the market adopt modern technological methods in their investment activities in order to keep up with recent financial developments and the impact of these activities on the performance of investment companies listed on the market. We also recommend that legislative bodies develop electronic payment systems to enhance financial security and protect investors' funds. Additionally, activating cooperation between agricultural companies and financial service providers is advised to benefit from their networks and reduce transaction fees.

The study suggests the following research topics: The impact of digital transformation on the performance of agricultural companies in the Iraqi financial market, and the impact of good governance on the performance of agricultural companies in the Iraqi stock market.

## Ethical approval

This is an original work of authors.

## Data Availability

The data supporting the findings of this study are available and published on the websites and official links listed below: http://www.isx-iq.net/isxportal/portal/homePage.html?currLanguage=en https://cbi.iq/static/uploads/up/file-176121620967824.pdf https://cbi.iq/static/uploads/up/file-170367704385390.pdf https://cbi.iq/static/uploads/up/file-166736775515698.pdf https://cbi.iq/static/uploads/up/file-176595635437154.pdf
