# Supplementary material for: The effectiveness of electronic payments on the market value of agricultural companies listed on the Iraq Stock Exchange
Source: F1000Res. 2026 Mar 5;15:357. [Version 1] doi: 10.12688/f1000research.177320.1 (PMC13054249; doi:10.12688/f1000research.177320.1)
Supplement: Peer review history

# Reviewer report: Reviewer response for version 1

**DOI:** 10.5256/f1000research.195524.r468989

**Published:** 2026-04-06

**Authors:** Kamil Picha

**Affiliations:** 1 University of South Bohemia, Ceske Budejovice, Czech Republic

- According to the abstract, the aim of the article is to enhance investment attractiveness and market performance. According to the Introduction, the aim of the article is to build a standard model that contributes to measuring fluctuations in the closing prices of agricultural companies listed on the Iraq Stock Exchange.
- However, the result and conclusion of the article is the confirmation of the hypothesis. It is therefore confusing for the reader to determine what the actual aim of the article is, and furthermore, neither of the stated aims appears to have been achieved.
- The presentation of results is limited to listing the individual results of the statistical analyses performed. I find the lack of a more detailed interpretation of the results, which could potentially lead to the fulfillment of the objective, to be a shortcoming.
- The authors also failed to provide a scientific discussion of the findings.
- The objectives and conclusions focus solely on the Iraqi market. It is not explained whether the situation in this market is entirely unique or whether the findings could potentially be applied to other markets as well.
- The practical implications are aimed at agricultural companies in Iraq and at policymakers in that country, and they appear to be beneficial. The theoretical implications are very brief and do not emphasize the expansion of the current state of knowledge. This is also related to the fact that a research gap was not identified at the outset. The only theoretical conclusion is a recommendation for further research.

Is the work clearly and accurately presented and does it cite the current literature?

Partly

If applicable, is the statistical analysis and its interpretation appropriate?

I cannot comment. A qualified statistician is required.

Are all the source data underlying the results available to ensure full reproducibility?

Yes

Is the study design appropriate and is the work technically sound?

Yes

Are the conclusions drawn adequately supported by the results?

Partly

Are sufficient details of methods and analysis provided to allow replication by others?

Yes

Reviewer Expertise:

Agricultural economics

I confirm that I have read this submission and believe that I have an appropriate level of expertise to confirm that it is of an acceptable scientific standard, however I have significant reservations, as outlined above.

# Reviewer report: Reviewer response for version 1

**DOI:** 10.5256/f1000research.195524.r468982

**Published:** 2026-04-03

**Authors:** Ashraf Bataineh

**Affiliations:** 1 Al-Balqa Applied University, Irbid, Jordan

Overall, the article is good. However, there are some points that need correction:

 A. Previous Studies: In this section, the researchers should include a more comprehensive review of previous studies, presenting them critically and analytically based on (variables, theories, or conflicting results). Its also necessary to clarify which studies demonstrated a positive relationship and which found an inverse relationship.

 B. Discussion: This study lacks a theoretical and empirical discussion of previous studies that support or contradict its hypotheses.

 C. Recommendations: In the results section, the researchers did not specify in their first recommendation which modern technological methods they recommend for use in investment companies.

 D. Conflict of Interest: No conflicts of interest were disclosed.

Is the work clearly and accurately presented and does it cite the current literature?

Yes

If applicable, is the statistical analysis and its interpretation appropriate?

I cannot comment. A qualified statistician is required.

Are all the source data underlying the results available to ensure full reproducibility?

Yes

Is the study design appropriate and is the work technically sound?

Yes

Are the conclusions drawn adequately supported by the results?

Partly

Are sufficient details of methods and analysis provided to allow replication by others?

Yes

Reviewer Expertise:

NA

I confirm that I have read this submission and believe that I have an appropriate level of expertise to confirm that it is of an acceptable scientific standard, however I have significant reservations, as outlined above.

## References

[1] : The effectiveness of electronic payments on the market value of agricultural companies listed on the Iraq Stock Exchange. *F1000Research* .2026;15: 10.12688/f1000research.177320.1 10.12688/f1000research.177320.1 PMC1305424941953065

# Reviewer report: Reviewer response for version 1

**DOI:** 10.5256/f1000research.195524.r465958

**Published:** 2026-03-27

**Authors:** Ilyas Ilyas Ilyas

**Affiliations:** 1 Universitas Halu Oleo, Kendari, South East Sulawesi, Indonesia

In general, the journal article is already good. There are only notes to be corrected:

 a. Introduction. In this section, data should be presented to support that the topic discussed is indeed an urgent problem to be researched.

 b. Method: This section should present the type, unit, and source of the data used.

 c. Discussion: In this section, theoretical and empirical reviews that support or contradict the research are not yet evident.

 d. Conclusion: In the Conclusion section, it is no longer necessary to present figures, as this has already been written in the previous section.

Is the work clearly and accurately presented and does it cite the current literature?

Yes

If applicable, is the statistical analysis and its interpretation appropriate?

I cannot comment. A qualified statistician is required.

Are all the source data underlying the results available to ensure full reproducibility?

Partly

Is the study design appropriate and is the work technically sound?

Yes

Are the conclusions drawn adequately supported by the results?

Yes

Are sufficient details of methods and analysis provided to allow replication by others?

Yes

Reviewer Expertise:

Economics

I confirm that I have read this submission and believe that I have an appropriate level of expertise to confirm that it is of an acceptable scientific standard.
